# The frequency of postdromal symptoms in patients suffering from migraine

**DOI:** 10.1186/1129-2377-14-S1-P117

**Published:** 2013-02-21

**Authors:** I Stanic, LJ Sretenovic

**Affiliations:** 1 Headache and Migraine Centre, Serbia and Montenegro

## Objective

To establish the percentage of postdromal symptoms in patients suffering from migraine headache according to sex, individual incidences and postdromal stage duration.

## Introduction

Migraine postdromes (MP) represent a set of various symptoms which persist for a certain period following a migraine headache attack. Many of those symptoms appear in the prodromal stage or during the headache phase. Most often, patients speak of losing appetite, experiencing nausea, muscular tension, diffuse headache, fatigue, hangover, change of mood or a congitive disturbance. The phase can last as long as 48 hours. Its pathophysiology is unknown, but it probably represents a phase of gradual recovery from an extreme neurological disturbance occurring during migraine. There have been very few studies in this field so far. Methodology: Research has been conducted on a sample of patients suffering from migraine with aura (MA) and those suffering from migraine without aura (M). The MP data have been obtained on the basis of interviews with 500 patients (F:M=400:100), aged between 18 and 40. 12% of the patients have experienced MA, whereas 88% of the patients have suffered from M. The number of patients for each subject of research has been also displayed in the form of percentages.

## Results

280 (70%) females and 55 (55%) male patients suffer from one or several postdromal symptoms. 480 (88%) patients of either sex experience postdromes lasting up to 24 hours, whereas 60 (12%) of patients experience postdromes lasting over 24 hours. The fatigue symptom is present in 360 (72%), diffuse headache in 165 (33%), a cognitive disturbance in 60 (12%), the loss of appetite in 35 (7%), hunger in 1 (0.2%), depression in 20 (4%), euphoria in 10 (2%), hangover in 55 (11%) and general weakness in 30 (6%).

## Conclusion

A large number of patients of either sex suffering from M or MA have postdromal symptoms lasting as much as 48 hours, which additionally aggravates their quality of life. It is therefore necessary to consider potential therapeutic protocols aimed at treating these complaints.

Key concepts of migraine postdrome:a qualitative study to develop a post-migraine questionnaire.
